# Effects of Endothelin-1 on Hepatic Blood Flow

**DOI:** 10.1155/1996/69047

**Published:** 1996

**Authors:** Kjetil Unneberg, Marianne Mjaaland, Elin Helseth, Arthur Revhaug

**Affiliations:** 1 Department of Surgery University Hospital of Tromosø Norway; 2 Department of Anaesthesiology University Hospital of Tromosø Norway

## Abstract

Endothelin-1 belongs to a family of potent vasoconstrictors, recently isolated from endothelial cells. Endothelin-1 has a variety of hepatic effects and hepatic clearance from the circulation is important. Elevated plasma concentrations of Endothelin-1 are found after orthotopic liver transplantation and in cirrhosis with ascites.

This study in piglets on hepatic bloodflow was designed to compare differences in effects between central venous and intraportal injection of endothelin-1, and to evaluate effects of repeated injections. Central venous injection of endothelin-1 caused a larger reduction in portal vein flow, while intraportal injection caused a larger increase in portal vein pressure. Repeated injections resulted in a reduction in portal vein flow and an increase in portal vein vascular resistance.


*HPB Surgery*, 1996, Vol. 9, pp.153–159
Reprints available directly from the publisher
Photocopying permitted by license only

© 1996 OPA (Overseas Publishers Association)
Amsterdam B.V. Published in The Netherlands
by Harwood Academic Publishers GmbH
Printed in Malaysia

### Effects of Endothelin-1 on Hepatic Blood Flow

KJETIL UNNEBERG[1], MARIANNE MJAALAND[1], ELIN HELSETH[2] and ARTHUR REVHAUG[1]

Departments of Surgery[1] and Anaesthesiology[2], University Hospital of Tromosø, Norway

Endothelin-1 belongs to a family of potent vasoconstrictors, recently isolated from endothelial cells. Endothelin-1 has a variety of hepatic effects and hepatic clearance from the circulation is important. Elevated plasma concentrations of Endothelin-1 are found after orthotopic liver transplantation and in cirrhosis with ascites.

This study in piglets on hepatic bloodflow was designed to compare differences in effects between central venous and intraportal injection of endothelin-1, and to evaluate effects of repeated injections. Central venous injection of endothelin-1 caused a larger reduction in portal vein flow, while intraportal injection caused a larger increase in portal vein pressure. Repeated injections resulted in a reduction in portal vein flow and an increase in portal vein vascular resistance.

KEY WORDS: Endothelin-1 hepaticblood flow portal vein flow portal vein pressure hepatic artery flow

Endothelin-1 was isolated from cultured porcine aortic endothelial cells in 1988[1]. The hemodynamic responses to endothelin are complex with a marked heterogeneity among species and different vascular beds. It is a potent vasoconstrictor in most vascular beds[2–5], but vasodilatory effects, interaction with endothelium-derived relaxing factors, dosage dependency, and regional differences have also been described[6–10]. In pigs, endothelin is released during endotoxin shock and asphyxia[11]. In patients, plasma levels of endothelin-1 are associated with the severity of sepsis[12], increased plasma levels are found after orthotopic liver transplantation[13], and in cirrhosis with ascites[14] have also been reported. In the liver, endothelin-1 diminishes microcirculation[15], and exhibits non-vascular effects like stimulated synthesis of mediators in Kupffer cells[16] and increased glucose production[17].

Endothelin-1 remains stable for up to one hour in blood, suggesting little enzymatic or spontaneous degradation. The rapid decrease of endothelin-1 levels from the circulation is mainly due to removal by parenchymal tissue[2,18]. Liver and lung have an important clearing function in several species, including man, while clearance in the porcine lung has been reported to be negligible[19].

The vasoconstrictor effects of endothelin-1 are more pronounced with extraluminal than intraluminal application, indicating a paracrine function[20]. The high fractional uptake and rapid clearance of endothelin-1 in several vascular beds further indicate that the site of origin is of importance. The liver contributes in maintenance of hemodynamic homeostasis by handling a multitude of vasoactive substances released into the portal blood stream in health and disease. Release of endothelin-1 into the portal vascular bed with first pass through the liver may have other effects on hepatic blood flow than a systemic release.

This study was designed to compare the effects of endothelin-1 on hepatic blood flow after central venous and intraportal injection, and to evaluate the effects of repeated injections. Changes in portal vein flow induces inverse changes in hepatic arteryflow. An attempt to distinguish these effect from systemic effects was made by concomitant recording of several other hemodynamic variables.

*Correspondence to*: Kjetil Unneberg, Dept. of Surgery, N-9038 University Hospital of Tromsø, Norway

### MATERIALS AND METHODS

The protocol was approved by the local Committee for Animal Experiments. Six Norwegian Landrace piglets weighing between 24 and 27 kg served as their own controls. The animals received a bolus injection of 10 nmol endothelin-1 (Novabiochem, Läufelfingen, Switzerland) centralvenously and intraportally. Flow in the portal vein, in the hepatic artery, and in the right renal artery, and pressure in the portal vein, in the superior caval vein, in the aorta, and in the pulmonary artery, were recorded continuously. The injections were separated by an interval of 30 minutes and the order of the injections was randomised. The injection time was 30 seconds. Three baseline periods were defined. The last five minutes before each injection were defined as Baseline 1 and Baseline 2, while Baseline 3 was defined from 30 to 35 minutes after the last injection. Basline values were used to evaluate the preinjection conditions and the effects of repeated injections. Peak and mean (for a single injection) deviation from the preceding baseline were used to compare effects of intraportal and centralvenous injections. When biphasic, peak effect was the amplitude with the larger absolute value. Mean effect was calculated from the area under the curve for 10 minutes after each injection. Integrals below zero level were defined as negative.

### *Surgical Procedure and Measurements*

The piglets were sedated in the Animal Department with ketamine (Ketalar®, Parke-Davis, S.A., Barcelona, Spain) 750 mg i.m. and 1 mg atropinsulphate (Atropin®, Hydropharma, Oslo, Norway). In the laboratory anaesthesia was induced with 5% isoflurane (Forene®, Abbott Laboratories Ltd, Queensborough, UK), 15 mg midazolam (Dormicum®, Roche, Basel, Switzerland), and 0.5 mg fentanyl (Leptanal®, Janssen Pharmaceutica, Beerse, Belgium). After endotracheal intubation, isoflurane was discontinued. The animals were ventilated (Servo Ventilator 900, Elema-Schönander, Stockholm, Sweden) With $O_2/N_2O$,6.51/min during surgery, and discontinuation of $N_2O$ at least 30 min before he experiments. FiO2 was 0.5. A uniform level of anaesthesis was maintained with a continuous infusion of ketamine (10 mg/kg/h during surgery, 5 mg/kg/h during the experiments) and midazolam 0.5 mg/kg/h. Blood temperature was maintained at 38±1°C with a heating pad.

Pulmonary artery pressure and central venous pressure were monitored by means of a 5 F Edwards Swan-Ganz catheter (Baxter Healthcare Corp., Santa Ana, CA, USA) inserted through the right jugular vein. A 7 F Edwards Swan-Ganz catheter was inserted into the aorta via the left carotid artery to monitor aortic pressure. The 5 F Swan-Ganz catheter was used to measure cardiac output. The animals were given heparin 200 IU/kg and a continuous infusion of Ringers acetate at a rate of 20 ml/kg/h. The bladder was drained via a cystotomy. Following a midline incision, limited dissection was performed to permit placement of perivascular flow probes around the portal vein (6 mm), the heptaic artery (3 mm), and the renal artery (2 mm). Care was taken to preserve the perivascular nerves. A central venous catheter (Secalon T, Viggo-Spectramed, Swindon, UK) was inserted into the portal vein.

Pressures were measured continuously with calibrated pressure transducers (Transpac 3, Abbott Critical Care Systems, North Chicago, III., USA). Flow was measured continuously with a ultrasonic transit-time flowmeter (Transonic Animal Research Flowmeters T208, Transonic Systems Inc. Ithaca, NY, USA). Pulsatile pressures and flows were monitored while mean flow signals (0.1 Hz second order Butterworth low-pass filtered) and mean pressure signals (0.05 Hz low-pass filtered) were recorded on a thermal chart recorder (Gould ES 2000, Gould Inc., Valley View, OI, USA). Heart rate was obtained from a digital display on the amplifier based on the pulsatile aortic pressure. Arterial and central venous pH, $P_{O2}$, $P_{CO2}$, base excess and $O_2$ saturation were analysed at the end of every baseline period by an ABL3 Acid Base Laboratory (Radiometer, Copenhagen, Denmark). A standard 3-lead electrocardiogram via subcutaneous electrodes was displayed on a EKG monitor (Diascope, Simonsen-Wedel, Copenhagen, Denkark) and a control heart rate obtained from the digital display.

### *Calculations*

Systemic vascular resistance, hepatic artery vascular resistance and portal vein vascular resistance were calculated from aortic pressure, central venous pressure, portal vein pressure cardiac output and hepatic artery flow.

### *Statistics*

Statistics were calculated on a Macintosh Quadra 950 using SuperANOVA (Abacus Concepts, Inc.,

Berkeley, CA, USA). ANOVA for repeated measures (multivariate approach, type III sum of squares) was used to evaluate differences in baseline trends for hemodynamic variables and blood gas analysis with time as a main effect. Contrast comparisons of means were used to evaluate pre injection differences in baseline values (Baseline 1 and Baseline 2). Paired *t*-tests were used to compare peak and mean effects of central venous and intraportal injection of endothelin-1. Values are presented as mean ± SEM. Significance level p = 0.05.

### RESULTS

All animals were included in the study. Due to technical difficulties (acoustic error), renal artery flow is missing in one animal.

### *Effects of Repeated Injections of Endothelin-1 on Baseline Values*

Baseline measurements are presented in table 1. The decline in portal vein flow was significant (p = .001). The difference between the pre-injection baseline levels were also significant (p = .03). The trend towards increased portal pressure was not significant (p = .10), but the increase in portal vein vascular resistance was significant (p = .03) with a trend towards difference in pre-injection values (p = .07). There was no effect on hepatic artery flow (p = .52), cardiac output (p = .35) and heart rate (p = .21). Aortic pressure increased significantly (p = .003), but systemic vascular resistance did not (p = .54). Central venous pressure decreased significantly (p = .003). Blood gas analysis revealed a non-significant trend towards decline in pH (p = .13).

### *Differences between Central Venous and Intraportal Injections of Endothelin-1*

Peak and mean effects of endothelin-1 after central venous and intraportal injections are presented in table 2. The reduction in portal vein flow was significantly larger after central venous injection (p = .0005 for peak reduction, p = .0006 for mean reduction). The increase in aortic pressure was significantly larger after central venous injection (p = .002 for peak increase, p = .005 for mean increase). Portal vein pressure was the only response significantly larger after intraportal injection of endothelin-1 (p = .002 for peak increase, p = .0005 for mean increase). The effect on hepatic

**Table 1** Baseline hemodynamic measurements

| *Hemodynamic variables* | *Baseline 1* | *Baseline 2* | *Baseline 3* | *p* |
|---|---|---|---|---|
| Hepatic artery flow (ml/min) | 115 ± 11 | 102 ± 18 | 101 ± 10 | 0.52 |
| Portal vein flow (ml/min) | 586 ± 13 | 535 ± 18 | 478 ± 30 | 0.001 |
| Renal artery flow (ml/min) | 86 ± 29 | 94 ± 29 | 92 ± 30 | 0.60 |
| Portal pressure (mm Hg) | 4.4 ± 0.4 | 5.5 ± 0.6 | 5.6 ± 0.3 | 0.11 |
| Central venous pressure (mm Hg) | 3.7 ± 0.3 | 3.4 ± 0.2 | 3.4 ± 0.3 | 0.003 |
| Pulmonary artery pressure (mm Hg) | 14.2 ± 1.6 | 15.8 ± 1.0 | 14.8 ± 1.1 | 0.43 |
| Aortic pressure (mm Hg) | 65 ± 2 | 74 ± 4 | 76 ± 4 | 0.003 |
| Cardiac output (L/min) | 2.2 ± 0.2 | 2.5 ± 0.2 | 2.4 ± 0.1 | 0.35 |
| Heart rate (per min) | 75 ± 4 | 83 ± 6 | 90 ± 8 | 0.21 |

**Table 2** Hemodynamic effects of Endothelin-1 after centralvenous and intraportal injection

| *Hemodynamic variables* | *Peak effect of C. V. inj.* | *Peak effect ofI.P. inj.* | *p* | *Mean effect of C. V. inj.* | *Mean effect of I.P. inj.* | *p* |
|---|---|---|---|---|---|---|
| Portal vein flow (mL/min) | –431 ± 79 | –156 ± 57 | 0.0005 | –293 ± 63 | –86 ± 28 | 0.0006 |
| Hepatic artery flow (mL/min) | 14 ± 120 | –30 ± 62 | 0.45 | 8 ± 32 | –19 ± 38 | 0.16 |
| Renal artery flow (mL/min) | –8 ± 68 | 10 ± 26 | 0.48 | –10 ± 31 | 6 ± 10 | 0.23 |
| Portal vein pressure (mm Hg) | 3.0 ± 1.5 | 6.5 ± 2.4 | 0.002 | 1.7 ± 0.8 | 4.3 ± 1.5 | 0.0005 |
| Central venous pressure (mm Hg) | 5.2 ± 5.4 | 1.2 ± 1.8 | 0.11 | 1.5 ± 2.7 | –0.5 ± 0.8 | 0.11 |
| Pulmonary artery pressure (mm Hg) | 7.3 ± 7.1 | 1.5 ± 0.5 | 0.11 | 2.8 ± 3.8 | –0.5 ± 0.5 | 0.10 |
| Aortic pressure (mm Hg) | 52 ± 18 | 7 ± 9 | 0.002 | 18 ± 8 | 2 ± 4 | 0.005 |

artery flow was less consistent, showing a mixture of vasoconstrictor and vasodilator effects. The effect on flow in the renal artery was similar to the effect on the hepatic artery.

Fig. 1 shows a thermal chart tracing from an animal that received the intraportal injection first and fig. 2 is from an animal that received the central venous injection first. The differences in effects are easily recognised in both figures.

### DISCUSSION

This study demonstrates that the effect of a bolus injection of endothelin-1 on portal blood flow is significantly less when injected into the portal vein compared to the effect of central venous injection. Thus, the larger amount of endothelin-1 that reaches the liver after an intraportal injection has less impact on portal vein flow than the smaller amount reaching the liver after a central venous injection. On the other hand, we found a larger increase in portal vein pressure after intraportal injection, reflecting the larger amount being presented to the portal vascular bed, creating a more massive vasoconstriction here, while correspondingly less reaches the systemic circulation. Both findings are in accordance with portal vein flow being mainly dependant on splanchnic inflow, while portal venous resistance influence portal pressure only, having little impact on portal vein flow[21]. Consistently, the peak reduction in portal vein flow precedes the peak increase in portal vein pressure (fig.1 and fig.2).

The differences in pre-injection baseline levels for portal vein flow and pressure have the same direction as the investigated differences between the two injection modes. Thus they tend to obscure rather than enhance any real differences.

The effect of endothelin-1 on hepatic artery flow is complex. In addition to the direct effects of endothelin-1, the enhanced reduction in portal vein flow induces a considerable vasodilator effect due to the hepatic artery buffer response[22]. When the central venous injection was given before the intraportal injection (fig.2), the effect was a marked reduction in hepatic arteryflow. However, when the central venous injection of endothelin-1 was given after the intraportal injection, the effect was a marked increase in hepatic artery flow (fig.1). We are unable to explain the presence of a "hepatic artery buffer response"- like effect only when the central venous injection was the second event, but occupation of receptors may change the balance between vasoconstriction (endothelin-1) and vasodilatation (hepatic artery buffer response). However, the same phenomenon is observed for the renal artery flow, indicating the occurrence of a different systemic effect rather than a local hepatic artery response. Little or no "hepatic artery buffer"-like responses were seen in connection with the smaller portal vein flow reductions after intraportal injections.

It is of hemodynamic importance not only how fast endothelin-1 is cleared, but also in which organ it is trapped. Wagner *et al.* showed pulmonary clearance to be a main cause of the short half-life of endothelin-1 in man, though splanchnic and pulmonary fractional extraction rates were comparable[19]. Increased pulmonary artery pressure was not a constant finding in our study, indicating a minor binding capacity in the porcine lung. A short half-life (77s) for endothelin-1 is reported in the pig[23]. The observed hemodynamic effects are clearly more long-lasting, reflecting the ability of endothelin-1 to bind tightly to its receptors.

There was a significant increase in baseline portal vein vascular resistance, and the significant downslide in baseline portal vein flow combined with unchanged cardiac output represents a redistribution at the expense of the gut. Since elevated plasma levels of endothelin-1 are found in ascites and sepsis, these effects may be of pathophysiological importance.

### CONCLUSION

Central venous injection of endothelin-1 causes a larger decrease in portal vein flow and a smaller increase in portal vein pressure than intraportal injection. The combined effect of repeated injections of endothelin-1 is a lasting reduction in portal vein flow and an increase in portal vein vascular resistance.

*Acknowledgements*

The skilful technical assistance of M-L Kjaereng, W. Gressnes and J. Bless is gratefully acknowledged. The study was supported by a grant from the Norwegian Council of Science.

### REFERENCES

1. Yanagisawa, M., Kurihara, H., Kimura, S., Tomobe, Y., Kobayashi, M., Mitsui, Y., Yzaki, Y., Goto, K., and Masaki, T (1988) A noval potent vasoconstrictor peptide produced by vascular endothelial cells. *Nature*, **332** 411–415.

2. Lüscher, T.F., Boulanger, C.M., Dohi, Y. and Yang, Z. (1992) Endothelium-Derived Contracting Factors. *Hypertension*, **19** 117–130.
3. Brain, S.D., Crossman, D.C., Buckley, T.L. and Williams, T.J. (1989) Endothelin-1: Demonstration of potent effects on the microcirculation of humans and other species, *Journal of Cardiovascular Pharmacology*, **13** (suppl 5) 147–149.
4. Clarke, J.G., Larkin, S.W., Benjamin, N., Keogh, B.E. and Chester, A. (1989) Endothelin-1 is a potent long-lasting vasoconstrictor in dog peripheral vasculature in vivo. *Journal of Cardiovascular Pharmacology*, **13** (suppl 5) 211–212.
5. Kiowski, W., Lüscher, T.F., Linder, L. and Bühler, F.R. (1991) Endothelin-1 induced vasoconstriction in man: Reversal by calcium channelblockade but not by nitrovasodilators or endothelium derived relaxing factor. *Circulation*, **83** 469–475.
6. Wright, C.E. and Frozard, J.R. (1988) Regional vasodilation is a prominent feature of the hemodynamic response to endothelin in spontaneously hypertensive rats. *European Journal of Pharmacology*, **155** 201–203.
7. Lüscher, T.F., Yang, Z., Tschudi, M., von Segesser, L., Stulz, P. and Boulanger, C.M. (1990) Interactions between endothelin and endothelium-derived relaxing factor in human arteries and veins. *Circulatory Research*, **66** (4) 1088–1094.
8. Leadly, R.J., Zhy, J.L. and Goetz, K.L. (1991) Effects of endothelin-1 and sarafotoxin S6b on regional hemodynamics in the conscious dog. *Americal Journal of Physiology*, **260** R 1210–1217.
9. Warner, T.D., Mitchell, J.A., de Nucci, G. and Vane, J.R. (1989) Endothelin-1 and endothelin-3 release EDRF from isolated perfused arterial vessels of the rat and rabbit. *Journal of Cardiovascular Pharmacology*, **13** (suppl 15) 85–88.
10. Boulanger, C.M. and Lüscher, T.F. (1990) Release of endothelin from the porcine aorta: Inhibition by endothelium derived nitric oxide. *Journal of Clinical Investigation*, **85** 587–590
11. Pernow, J., Hemsen, A., Hallén, A. and Lundberg, J.M. (1990) Release of endothelin-like immunoreactivity in relation to neuropeptide Y and catecholamines during endotoxin shock and asphyxia in the pig. *Acta Physiologica Scandinavica*, **140** 311–322.
12. Pittet, J.F., Morel, D.R., Hemsen, B.S., Gunning, K., Lacroix, J.S., Suter, P.M. and Lundberg, J.M. (1991) Elevated plasma endothelin-1 concentrations are associated with severity of illness in patients with sepsis. *Annals of Surgery*, **213**(3) 261–264.
13. Lerman, A., Click, R.L., Narr, B.J., Wiesner, R.H., Krom, R.A.F., Textor, S.C. and Burnett, J.C. Jr. (1991) Elevation of plasma endothelin associated with systemic hypertension following orthotopic liver transplantation. *Transplantation*, **51**(3) 646–650.
14. Asbert, M., Gines A., Gines, P., Jimenez, W., Claria, J., Salo, J., Arroyo, V., Rivera, F. and Rodes, J. (1993) Circulating levels of endothelin in cirrhosis. *Gastroenterology*, **104**(5) 1485–1491.
15. Kurihara, T., Akimoto, M., Kurokawa, K., Ishiguro, H., Niimi, A., Maeda, A. and Sigemoto, M. *et al.* (1992) Relationship between endothelin and thromboxane A2 in the rat liver microcirculation. *Life Sciences*, **51**(26) PL281–285.
16. Gandhi, C.R., Harvey, S.A. and Olson, M.S. (1993) Hepatic effects of endothelin: metabolism of endothelin-1 by liver-derived cells. *Archives of Biochemistry and Biophysics*, **305**(1) 38–46.
17. Roden, M., Vierhapper, H., Liener, K. and Waldhäusl, W. (1992) Endothelin-1 stimulated glucose production in the isolated perfused rat liver. *Metabolism*, **41** 290–295.
18. Shiba, R., Yanagisawa, M., Miyauchi, T., Ishii, Y., Kimura, S., Uchiyama, Y., Masaki, T. and Goto, K. (1989) Elimination of intravenously injected endothelin-1 from the circulation of the rat. *Journal of Cardivascular Pharmacology*, **13** (suppl 5) 98–101.
19. Wagner, O.F., Vierhapper, H., Gasic, S., Nowotny, P. and Waldhäusl, W. (1992) Regional effects and clearance of endothelin-1 across pulmonary and splanchnic circulation. *European Journal of Clinical Investigation*, **22** 277–282.
20. Pohl, U. and Busse, R. (1989) Differential vascular sensitivity to luminally and adventitially applied endothelin-1. *Journal of Cardiovascular Pharmacology*, **13** (suppl 5) 188–190.
21. Greenway, C.V. and Lautt, W.W. (1989) Hepatic circulation. In *Handbook of physiology, The Gastrointestinal System*; edited by J.D. Wood vol. 1(2), pp. 1519–1564. New York: Oxford University Press
22. Lautt, W.W. (1985) Mechanism and role of intrinsic regulation of hepatic arterial blood flow: the hepatic artery buffer response. *American Journal of Physiology*, **249** G549–G556.
23. Pernow, J., Hemsen, A. and Lundberg, J.M. (1989) Tissue specific distribution, clearence and vascular effects of endothelin in the pig. *Biochemical and Biophysical Research Communications*, **161** (2) 647–653

